# Deep Eutectic Solvents Mediated Extraction of a Pectin Polysaccharide from Processed Sweet Potato By-Products: Optimization and Characterization Studies

**DOI:** 10.3390/foods15020388

**Published:** 2026-01-21

**Authors:** Wenting Zhang, Ke Liu, Jian Sun, Xiaoxue Liang, Juntao Guo, Qiang Li, Chanmin Liu

**Affiliations:** 1 School of Life Sciences, Jiangsu Normal University, Xuzhou 221116, China; 2 Xuzhou Institute of Agricultural Sciences in Jiangsu Xuhuai District/Key Laboratory of Biology and Genetic Breeding of Sweet Potato, Ministry of Agriculture and Rural Affairs/Sweet Potato Research Institute, Chinese Academy of Agricultural Sciences (CAAS), Xuzhou 221131, China

**Keywords:** sweet potato residue, pectin, deep eutectic solvents, characterization, anti-inflammatory activity

## Abstract

In this study, a pectin polysaccharide named DESP was extracted using a deep eutectic solvent (DES) from sweet potato residue (SPR) and the extract was optimized through response surface methodology (RSM). The DESP, based on choline chloride–urea (ChCl-Ur), was characterized for yield, molecular weight (*M*w), and monosaccharide composition. Fourier-transform infrared spectroscopy (FT-IR), X-ray diffraction (XRD), ^1^H-nuclearmagnetic resonance (^1^H-NMR), and scanning electron microscopy (SEM) were used to analyze the structure. Optimal extraction conditions for DESP were ChCl-Ur in a molar ratio of 1:2, water content of 75 wt.%, extraction time of 125.7 min, extraction temperature of 83.2 °C, and a liquid-to-solid ratio of 37.0 mL·g^−1^. The optimized extraction yield was 5.6% ± 0.09%, which was 2.4 times higher than that of hot-water-extracted sweet potato pectin (HWSP, 2.32%). The monosaccharide analysis revealed that galacturonic acid (GalA) was the most abundant saccharide, followed by glucose (Glc), galactose (Gal), arabinose (Ara), and rhamnose (Rha). The *M*w of DESP was 20.90 kDa, which was lower than that of HWSP and HASP. In addition, DESP exhibited certain anti-inflammatory activity.

## 1. Introduction

Deep eutectic solvents (DESs), whose physicochemical properties are similar to ionic liquids, have the characteristics of low volatility, degradability, high thermal stability, accessibility, customizability, and environmental friendliness [1]. They are formed by the thermal mixing of two or more components, including hydrogen bond donors (HBDs) and hydrogen bond acceptors (HBAs), through hydrogen bonding. The melting temperature of the formed DESs is lower than the melting temperature of the individual compositions and is typically less than 100 °C. DESs have better safety profiles and have less negative impact on the environment than traditional organic solvents [2]. As promising green solvents, DESs have been applied to extract some biopolymers such as polyphenols [3], alkaloids [4], and proteins [5]. Due to the selective solubilization of polysaccharides, DESs are particularly well suited for polysaccharide extraction, including the extraction of date pomace polysaccharides [6], quince peels polysaccharides [7], sweet tea polysaccharides [8], and Lentinus edodes polysaccharides [9,10].

Take the research on the extraction of pectin polysaccharides as an example; DESs can be used as green alternatives to traditional solvents such as hot water or acid extraction methods, which have disadvantages such as long processing time and high energy consumption [11]. Studies have shown that DES has a greater interaction with plant materials, thus significantly increasing the extraction rate of pectin. Thus, DES has been widely used in the extraction of pectin from fruits and other plants. For instance, researchers utilized DES to assist in extracting pectin from *Ficus carica* Linn. (fig) peels. Under the optimal preparation process, the maximum yield of pectin (239.6 g/kg) was twice that of the traditional hot water extraction method. Moreover, the pectin extracted based on DES possesses superior physicochemical properties and biological performance, including high uronic acid content, solubility, dispersibility, emulsifying performance, and antioxidant activity [12]. In another study, researchers used DES to extract high-quality pectin from grapefruit (*Citrus paradisi*) peels. Under acidic conditions (pH 2.0), the yield (36.47%) of pectin extracted using DES composed of betaine-citric acid was significantly higher than that extracted with HCl (8.8%). More importantly, the pectin extracted using DES showed better emulsifying performance than that extracted in hydrochloric acid [13]. Similarly, Lin et al. [14] extracted pectin from the fruit of *Clausena lansium* (Lour.) Skeels using DES and compared the basic and functional characteristics of pectin extracted by DES (D-CP) and pectin extracted by water (W-CP). It was found that D-CP under the optimal extraction conditions exhibited an excellent yield (18.09%), and also showed better solubility, foam stability, and antioxidant activity than W-CP. These studies all indicate that DES has been proven to be an effective solvent for the efficient extraction of bioactive pectin from plant species.

Since the DES system contains more hydrogen bonds, providing additional interaction with the plant material, it therefore solubilizes more polysaccharides relative to acidic or alkaline solvents [15]. Nevertheless, the type of DES, extraction temperature, and extraction time can still affect the yield and composition of polysaccharides. A certain range of water content in DESs can effectively regulate the viscosity and increase the mass transfer efficiency of active ingredients. However, a higher water content can break hydrogen bonds, destroying their structure and efficacy [16]. Temperature has a large effect on the viscosity and diffusion coefficient of DESs. Therefore, it can directly affect the solubility of active substances. Higher extraction temperature can also break glycosidic bonds of polysaccharides and affect their biological activity; thus, extraction temperature should be optimized. Additionally, the dissolution rate of active ingredients generally reaches equilibrium after a certain period of time, limiting the extraction time.

Sweet potato (*Ipomoea batatas*. L) is one of the five most important food crops in the world, and China is the largest producer, with an annual output of more than 53 million tons [17,18]. Sweet potato is mainly used to process starch, leading to a large amount of SPR by-product, which is approximately 4.5 times the amount of raw sweet potato [19]. However, the accumulation of SPR with high moisture content promotes fermentation and the release of methane, contributing to environmental pollution [20]. In recent decades, methods for generating high-value-added products from the by-products of agricultural product processing are highly sought after, and dietary fiber [21], polyphenols [22], and pectin [23,24] are high-demand, functional ingredients. SPR is rich in dietary fiber-based nutrients, containing up to 20% pectin, making it a non-negligible material for pectin extraction [25,26]. Therefore, SPR is an under-utilized resource with high value potential.

In our previous research, we extracted four pectin polysaccharides from SPR using conventional chemical solvents [27]. However, the traditional method of extracting pectin from SPR has a low yield and is not sufficient to meet the needs of industrial production. Therefore, there is an urgent need to explore an effective and environmentally friendly method for preparing pectin from SPR. At present, systematic research on extracting pectin from SPR using DES has not been reported. To develop an effective method for extracting active pectin from SPR, we determined the optimal DES extraction process by combining single-factor experimental methods and BBD methods. The pectin extracted from DES (DESP) was characterized by yield analysis, monosaccharide composition, molecular weight (*M*w) distribution, degree of esterification (DE), Fourier transform infrared spectroscopy, XRD, and electron microscopy analysis, and was compared with pectin extracted from hot water (HWSP). In addition, the anti-inflammatory properties of DESP were also studied to evaluate the effect of DES extraction on the biological activity of SPR pectin. This study may propose a new extraction strategy to promote the utilization of SPR pectin in industrial applications.

## 2. Materials and Methods

### 2.1. Materials and Reagents

Sweet potato residue was provided by Shandong Sishui Lifeng Food Co., Ltd., Jining, China. Lipopolysaccharide (LPS, from *E. coli* O55: B5), dexamethasone (DEX), and phorbol 12-myristate 13-acetate (PMA) were purchased from Sigma-Aldrich (St. Louis, MO, USA). Enzyme-linked immunosorbent assay (ELISA) kits were obtained from Proteintech (Wuhan, China).

### 2.2. Preparation and DES Type Screening

According to Chen et al. [28], DESs were obtained by mixing the two components, hydrogen bond acceptors (HBA) and hydrogen bond donors (HBD), with magnetic stirring in a certain condition (80 °C~85 °C, 2–4 h) until a clear homogeneous liquid was formed. Six different types of DESs were prepared, as shown in Table 1, including choline chloride-1,3-propylene glycol, choline chloride-glycerol, choline chloride-citric acid, potassium carbonate-glycerol, choline chloride- urea, and choline chloride-1,4-butanediol with 60 wt.% water to decrease the viscosity. Taking the extraction yield as an index, HBA and HBD with relatively high extraction efficiency were further screened out.

### 2.3. Extraction of DESP by DESs

Based on our previous method [27] and pre-experiments, we explored a method for extracting crude SPR pectin using DES. Briefly, 50 g of SPR powder was soaked in 500 mL of anhydrous ethanol and stirred at room temperature for 1 h to remove impurities. Subsequently, under appropriate conditions (as described in Section 2.4), SPR pectin was extracted using DESs. The extracted solution was then centrifuged (5000 rpm, 20 min), and the supernatant was retained and concentrated to one-fifth its original volume. Next, the extraction solution was enzymatically treated in a neutral environment (pH 6.5–7) for 1.5 h at 95 °C using a 0.5% high-temperature-resistant α-amylase (CAS: 9000-90-2; activity: 10,000 u/g) to remove starch macromolecules. After another centrifugation (5000 rpm, 15 min), the pectin crude extract was precipitated with anhydrous ethanol; washed sequentially with 50%, 70%, and 95% ethanol; and filtered under reduced pressure. The crude pectin was then dissolved in water, dialyzed (5000 Da MWCO) for 72 h (changing the water every 3 h), and freeze-dried to obtain the final pectin product. The moisture content of dried pectin was 5%. The yield calculation of pectin is shown in Formula (1). “m1” and “m2”, respectively, refer to the weight of extracted dried pectin and the weight of SPR.

(1) $$Yield\;(\%)\;=\;\frac{m1}{m2}\;\times \;100\%\;\;\;$$

### 2.4. Single-Factor Experiment Design

The single-factor experimental method was used to analyze the effects of water content (15 wt.%, 30 wt.%, 45 wt.%, 60 wt.%, and 75 wt.%), extraction time (30 min, 60 min, 90 min, 120 min, and 150 min), extraction temperature (20 °C, 40 °C, 60 °C, 80 °C, and 100 °C), and solid–liquid ratio (10 mL·g^−1^, 15 mL·g^−1^, 20 mL·g^−1^, 25 mL·g^−1^, 30 mL·g^−1^, 35 mL·g^−1^, 40 mL·g^−1^, and 45 mL·g^−1^) on the extraction yield.

### 2.5. Response Surface Methodology Design

RSM was used to calculate the effects of 4 independent variables at 3 levels on the extraction yield. A Box–Behnken design (BBD) was employed for designing the experiments [29]. Each variable is encoded into three levels: −1, 0, and 1 (Table 2). RSM was applied to the random experimental data using the commercial statistical package Design-Expert version 8.0.6. Based on the preliminary experimental results, the entire design consists of 29 random experimental points (Table 3).

### 2.6. Characterization of DESP

#### 2.6.1. *M*w Determination and Monosaccharide Composition Analysis

According to the previous method, the *M*w and monosaccharide composition analysis of the pectin were conducted based on a high-performance gel permeation chromatography (HPGPC) system (Shimadzu, Kyoto, Japan) [30]. Please refer to the Supplementary documents for detailed method descriptions.

#### 2.6.2. FT−IR and NMR Analysis

In brief, the dry pectin was mixed with potassium bromide powder, compressed into tablets, and scanned using FT−IR (Cary 670 FT−IR, Agilent, Santa Clara, CA, USA). Based on the FT−IR spectrum, the degree of esterification (DE) was evaluated as the percentage of the peak area at 1741 cm^−1^ (COO−R) to the sum of peak areas of the two peaks at 1741 cm^−1^ (COO−R) and 1610 cm^−1^ (COO−) [31].

The dried pectin sample was dissolved in a suitable solvent, and the ^1^H−NMR was scanned using a Quantum−I Plus 600 MHz spectrometer (QOne, Dietlikon, Switzerland).

#### 2.6.3. SEM Analysis

A field emission scanning electron microscope (S-48001 FESEM, Hitachi High-Technologies Corporation, Tokyo, Japan) was used to scan the sample.

#### 2.6.4. XRD Analysis

An X-ray diffractometer (XRD, Bruker, D8 Advance A25, Karlsruhe, Germany) with a Cu Kα radiation source was used to detect the sample.

### 2.7. Determination of In Vitro Anti-Inflammatory Activities

According to our previous method [27], the immortalized bone marrow-derived macrophage (iBMDM) cells were cultivated overnight in a 96-well plate and the cytokines in the culture supernatant were assessed by ELISA kits (Abcam, Shanghai, China).

### 2.8. Statistical Analysis

All data were expressed as mean ± standard deviation of triplicate measurements. Differences were assessed by a one-way analysis of variance with Duncan’s multiple range test. Analyses were performed using GraphPad Prism version 10.1, and graphs were created using Origin 2021 software. Design Expert version 8.0.6 was used for the response surface experimental design and statistical analysis.

## 3. Results and Discussion

### 3.1. DES Type and Molar Ratio Screening

**Figure 1:**
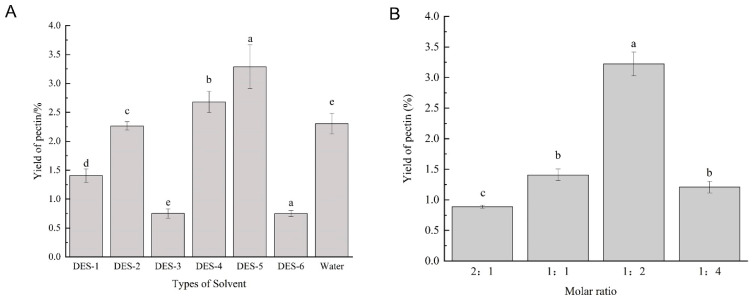
Comparison between six DESs and water extraction yield for sweet potato pectin (**A**). Comparison between molar ratio of choline chloride and urea (**B**). Different lower-case letters show a significant difference among different extraction methods (*p* < 0.05).

Due to the different types of DES, their chemical properties, such as viscosity, polarity, and solubility, also vary, which affects the yield of pectin [32,33]. With the conditions of a water content of 60 wt. %, an extraction time of 90 min, an extraction temperature of 80 °C, and a solid–liquid ratio of 30 mL·g^−1^ fixed, the pectin extraction efficiency of six different DESs was evaluated. The yield varies considerably depending on the types of DESs. DES-5 shows the highest yield of 3.3% (Figure 1A), which may be due to its strong electrostatic interactions with pectin. The variability in extraction efficiency may be due to differences in pH, polarity, viscosity, solubility, and surface tension between different DESs [34]. Therefore, DES-5 is chosen as the extraction solvent for the subsequent optimization experiments.

The effect of the molar ratio of choline chloride to urea on the extraction yield of pectin was further investigated. As shown in Figure 1B, as the proportion of urea increases, the extraction yield also increases. The yield reaches the maximum (3.22%) when the molar ratio of choline chloride to urea is 1:2. However, as the molar ratio changes from 1:2 to 1:4, the yield decreases. This is attributed to the low proportion of choline chloride in the DES system, which limits the hydrogen bonding interaction between pectin and DES components [35,36]. Thus, the optimal molar ratio is set at 1:2.

### 3.2. Single-Factor Experiments

**Figure 2:**
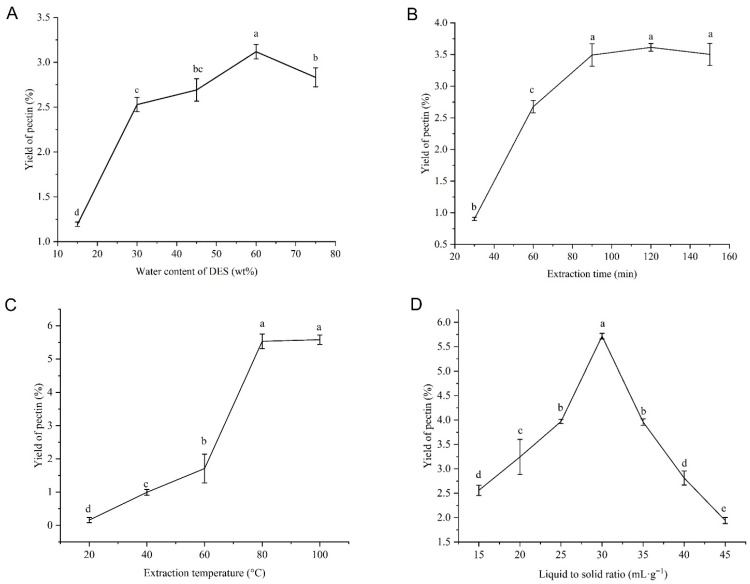
The influence of four factors of DES-5 on pectin yield in a single-factor experimental design. (**A**) The influence of water content of DES on the yield of pectin. (**B**) The influence of extraction time on the yield of pectin. (**C**) The influence of extraction temperature on the yield of pectin. (**D**) The influence of liquid to solid ratio on the yield of pectin. Different lowercase letters show a significant difference among different extraction methods (*p* < 0.05).

The yields of the pectin increase significantly when the water content of DES-5 increases from 15 wt.% to 30 wt.% (Figure 2A). When the water content of DES-5 continues to increase to 45 wt.%, the yield slows. At 60 wt.%, the yield reaches its peak and then decreases. Appropriate water content reduces the high viscosity of DES-5, making it easier to penetrate plant cells and extract pectin. However, excessively high content leads to a decrease in intermolecular forces between choline chloride and urea, as well as a reduction in the interaction between DES and pectin. This, in turn, results in a decrease in yield [37]. Hence, the optimal water content for DESP extraction ranges from 45 wt.% to 75 wt.%.

Extraction time also greatly impacts the extraction yield of pectin. The raw materials and solvents must be in contact for sufficient time to achieve equilibrium between the pectin concentration in sweet potato residue tissue and the pectin concentration in the solvent. In our study, Figure 2B displays the increase in pectin yield from 30 to 120 min. The yield slightly decreases when the extraction time exceeds 120 min. It indicates that at high temperatures, all pectin in the sample reaches equilibrium within 120 min, and extending the extraction time does not help improve the yield [38]. This may be due to the hydrolysis of pectin over time [39]; therefore, the extraction time is controlled within 150 min.

The maximum yield of pectin can be obtained at 80 °C (Figure 2C). Once the temperature continues to rise, there is no significant increase in pectin yield. Due to the viscosity of the DES system decreasing to a certain extent with increasing temperature, it is beneficial for DES to dissolve pectin components from cells. Importantly, excessive extraction temperature leads to the thermal degradation of polysaccharide components [40]. Therefore, the optimal extraction temperature for pectin ranges from 60 °C to 100 °C.

The liquid-to-solid ratio also has a significant impact on the yield of pectin. Figure 2D shows that the yield of pectin is the highest when the liquid-to-solid ratio is 30 mL·g^−1^. The reason for the increase in yield is the increase in the proportion of solvent, which allows more solvent to penetrate into the cell tissue and come into contact with the pectin, thereby improving the diffusion rate [41]. When the liquid–solid ratio is lower than 15 mL·g^−1^, the water absorption and swelling of sweet potato residue lead to the high viscosity of the solution system, hindering sufficient stirring and the extraction of pectin. In contrast, an excessively high liquid–solid ratio decreases the yield significantly. Therefore, considering the solvent cost, a solid–liquid ratio range of 24–45 mL·g^−1^ is chosen.

### 3.3. Box–Behnken Design and Analysis

#### 3.3.1. Analysis of the Quadratic Regression Model

The experimental design matrix and the corresponding results of RSM experiments are depicted in Table 3. The predicted response (Y) for the yield is fitted in the equation below:

Y(%) = 5.75 + 0.30A + 0.30B + 0.32C − 0.19D + 0.18AB − 0.04AC + 0.51AD − 0.32BC − 0.63BD + 0.24CD − 0.41A^2^ − 0.82B^2^ − 0.83C^2^ − 0.63D^2^

where Y is the yield of sweet potato pectin and A, B, C, and D represent the water content of DES-5, extraction time, extraction temperature, and liquid-to-solid ratio, respectively.

As shown in Table 4, the predicted model fit is verified, with an F-value of 41.16 and a *p*-value of 0.0001. The *p*-value for lack of fit is 0.6534 (>0.05), which is not significant and indicates the model is well established. The coefficient of determination (R^2^) for the model fit is 0.9763, implying that 97.63% of the total variation in response can be elucidated. The values of R_Adj_^2^ (0.9526) and R_pred_^2^ (0.8968) represent the true relationship of these parameters and the significance of the model. The C.V.% (3.53%) indicates high reliability, and the precision (21.89%) indicates that the effect is precise, thus suggesting a good fit to the experimental results.

From Table 4, it can be seen that the order of factors affecting pectin yield is as follows: extraction temperature (C) > extraction time (B) > water content (A) > liquid-to-solid ratio (D). The water content (A), extraction time (B), extraction temperature (C), and liquid-to-solid ratio (D) all have significant effects on the yield of pectin (*p* < 0.001). Of the interaction terms, AD, BD, BC, and CD have significant effects on the yield of pectin (*p* < 0.001, *p* < 0.05 for CD). The quadratic terms all have a certain effect on the yield of pectin (*p* < 0.001). The maximum yield (5.73%) was achieved with conditions of water content of 75 wt.%, extraction time of 125.66 min, extraction temperature of 83.17 °C, and a liquid-to-solid ratio of 36.95 mL·g^−1^. However, considering the convenience of operation, the validation experiment is conducted under the following conditions: water content of 75 wt.%, extraction time of 125 min, extraction temperature of 83 °C, and liquid-to-solid ratio of 37 mL·g^−1^. The actual extraction yield of DESP extracted by DES is 5.6% ± 0.09% (n = 3), and it is in agreement with the predicted value (5.6%).

#### 3.3.2. Analysis of Response Surfaces

**Figure 3:**
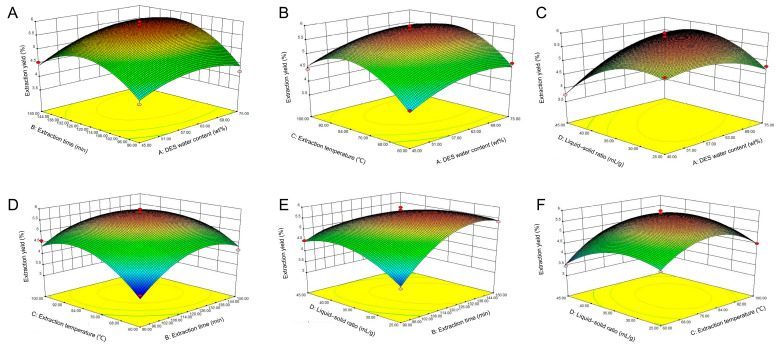
Three-dimensional response surface plots of DES extraction. (**A**–**F**) Interactions between water content, extraction time, extraction temperature, and liquid–to–solid ratio, respectively.

**Figure 4:**
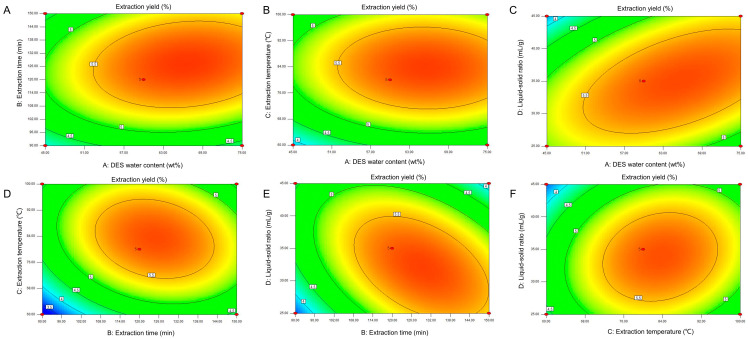
Two-dimensional contour plots of DES extraction. (**A**–**F**) Interactions between A, B, C, and D, respectively.

Regression models can be visualized through response surfaces and contour maps, which allow an intuitive understanding of the impact of various factors on experimental results [42]. The response surface map and contour plots of the extraction yields not only analyze the impact of multiple independent variables on response values but also reflect the sensitivity of response values to changes in different independent variables. A steeper response surface curve leads to a greater elliptical eccentricity in the contour line, indicating there is a significant interaction [43]. In Figure 3 and Figure 4, the interaction effects between B and D, A and D, and B and C are significant, which is corroborated by the *p*-values of interaction coefficients (BD, AD, and BC) shown in Table 4.

### 3.4. Structural Characterization of DESP

#### 3.4.1. The Yields and *M*w

As shown in Table 5, the DES method provides a higher extract yield (5.6%), while the conventional extraction method presents a lower yield (2.3% or 2.1%) [27]. This indicates that DES is efficient at increasing pectin polysaccharide extraction [44]. Similar findings were reported by Nie et al. [45] and Saravana et al. [46]. An interpretable reason may be that the richer hydrogen bonds between DES and pectin polymer result in higher viscosity and better permeability of the solution system, which is beneficial for the migration of pectin [47].

**Figure 5:**
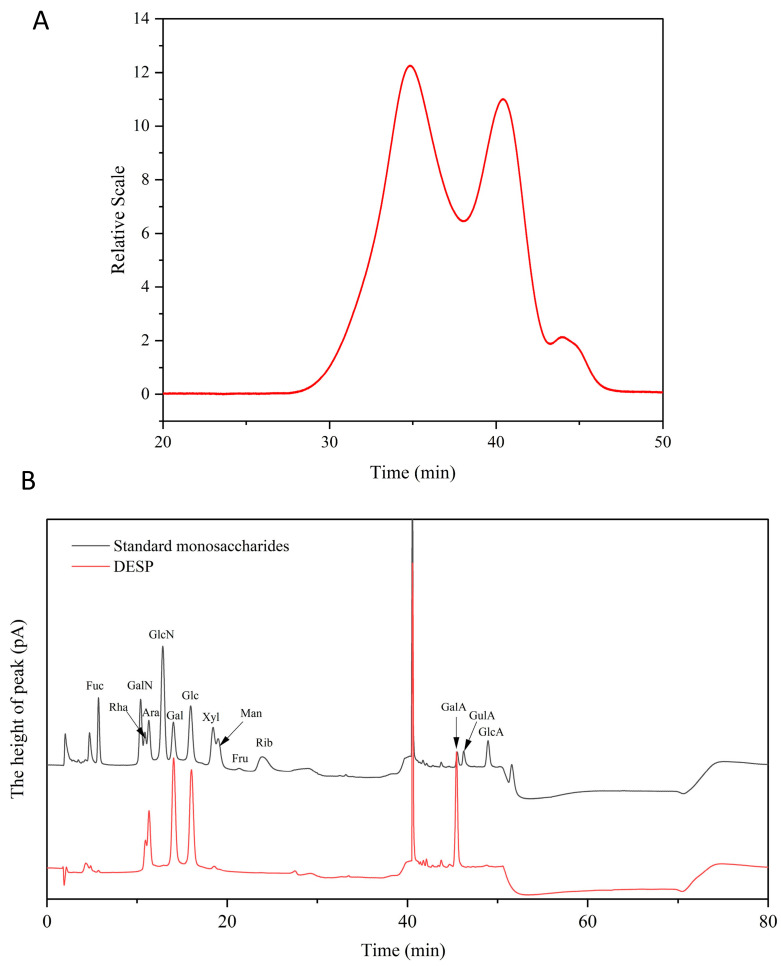
HPGPC elution curve (**A**) and HPLC chromatograms of standard monosaccharides and DESP (**B**).

As shown in Table 5 and Figure 5A, the *M*w of DESP, hot water extraction (HWSP), and hydrochloric acid extraction (HASP) of sweet potato pectin are 20.90 kDa, 58.41 kDa, and 69.81 kDa, respectively, indicating that the *M*w of pectin extracted by DES is significantly lower than that extracted by hot water or hydrochloric acid. The possible reason for this is that the hydrogen bonding of DES leads to the degradation of pectin [48].

#### 3.4.2. Monosaccharides Composition

As shown in Figure 5B and Table 6, the composition of DESP comprises galacturonic acid (GalA, 47.07%), glucose (Glc, 25.63%), galactose (Gal, 17.9%), arabinose (Ara, 12.1%), rhamnose (Rha, 4.13%), and xylose (Xyl, 1.07%). Compared to HWSP and HASP, the content of Gal in DESP decreases, and it may be due to DES extracting more cellulose and hemicellulose components. Similar reports have also been found by Turan et al. [49], whose research results indicate that the extract from orange peel using DES contained a high amount of total sugars compared to commercial pectin, which may have contributed to high yields. In addition, the molar ratio of (Ara + Gal)/Rha represents the degree of branching in the Rhamnogalacturonan-I (RG-I) region [50]. From Table 6, the (Ara + Gal)/Rha ratios of DESP, HWSP, and HASP are 7.26 ± 0.57, 4.02 ± 0.05, and 2.25 ± 0.05, respectively, indicating that DESP has a higher degree of branching and longer side chains. The factors involved in the extraction process, such as solvent type, time, and temperature, have an impact on the structure of natural pectin [51]. In addition, according to the calculation, the DE is 26.15%. Hosseini et al. [52] reported that the pH of the extraction solvent is related to DE, and the lower the pH of the solvent, the lower the DE.

**Figure 6:**
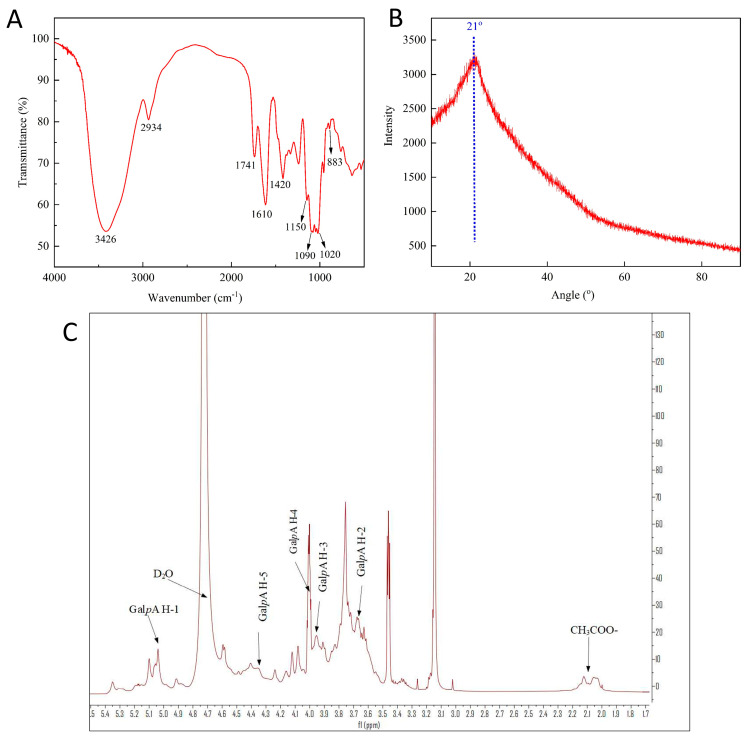
The FT−IR spectra (**A**), XRD spectra (**B**), and ^1^H NMR spectra (**C**) of DESP.

FT-IR is used to analyze the chemical bonds and functional groups of DESP. As shown in Figure 6A, the intense, broadly stretched peak near 3426 cm^−1^ represents the O–H stretching vibrations, and the small peaks at approximately 2934 cm^−1^ are attributed to the C−H stretching and bending vibrations of free sugars [52]. The peaks at approximately 1741 and 1610 cm^−1^ are assigned to the stretching vibrations of −COOR and −COO− [53], respectively, which might indicate the presence of uronic acid. In addition, the peaks at 1150 cm^−1^, 1090 cm^−1^, 1020 cm^−1^, and 883 cm^−1^ represent the backbone signals of the pyranose ring and α-D-glucopyranose, respectively [54]. In the XRD pattern (Figure 6B), a broad and strong characteristic peak at 21° is detected, indicating that the pectin component is an amorphous substance [55]. Figure 6C shows the ^1^HNMR spectra of DESP. Most of the signals appear between 2.0 and 5.4 ppm. The signals at 5.03, 3.67, 3.95, 4.00, and 4.35 ppm correspond to H−1, H−2, H−3, H−4, and H−5 of GalA protons, respectively [56]. The signal near 2.08 ppm is attributed to the methylene of GalA [57].

#### 3.4.3. Scanning Electron Microscopy

**Figure 7:**
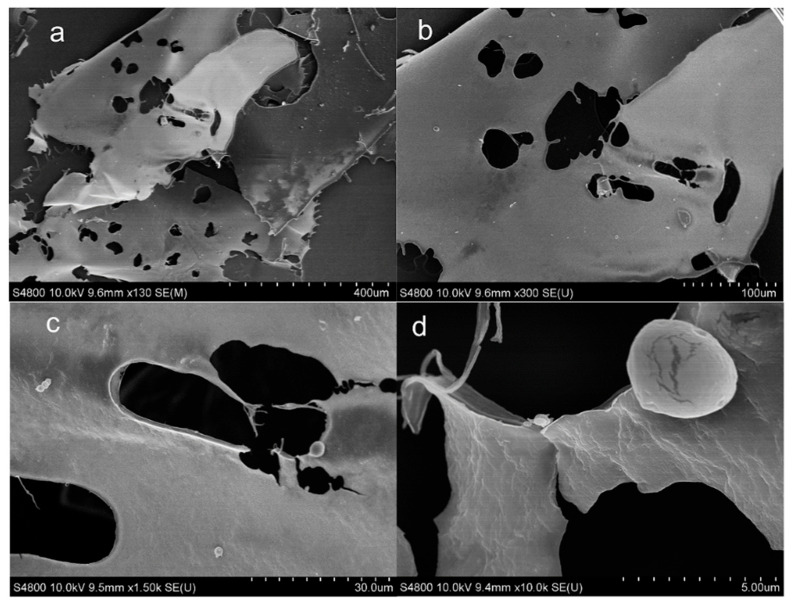
The SEM images of DESP ((**a**): 130×; (**b**): 300×, (**c**): 1500×; (**d**): 10,000×).

SEM is a commonly used method to explore the surface microstructures of pectin. As shown in Figure 7, the microstructure of DESP is a thin sheet with many holes, and there are several spherical particles attached to the surface, which is different from traditional extraction methods [27]. There is a large network structure composed of hydrogen bonds between the pectin components extracted by DES, which makes the pectin surface more fragile. The surface also has a larger contact area with the solvent. Therefore, DES increases the pectin dissolution rate and yield, which is consistent with the findings of Fu et al. [43].

### 3.5. Effect of Reuse of DES on the Yield of DESP

**Figure 8:**
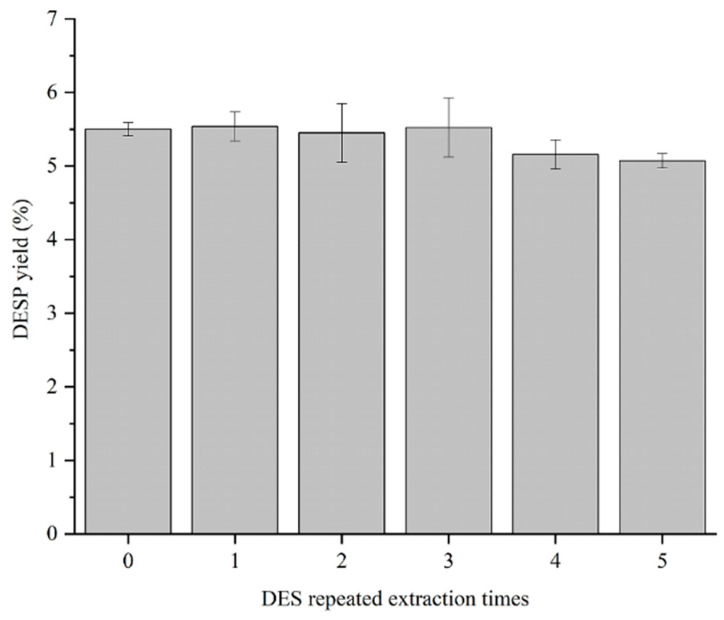
Effect of repeated use of DES on the yield of DESP.

DES can be recovered from sweet potato pectin extract. As shown in Figure 8, the overall trend of extraction yield shows a slight decrease with the yields of 5.61%, 5.66%, 5.45%, 5.52%, 5.16%, and 5.07%, respectively, from 0 to 5 times of repeated use. The recycling studies demonstrate the good recycling and reusing characteristics of this DES.

### 3.6. In Vitro Anti-Inflammatory Activity

#### 3.6.1. iBMDM Cell Viability

**Figure 9:**
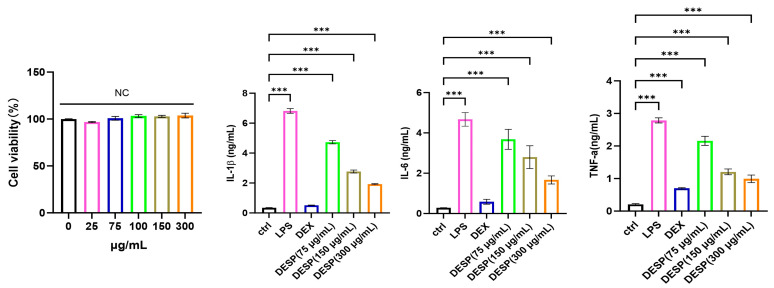
Cell viability of DESP on iBMDM cells in the presence of LPS and the effects of pectin on cytokine secretion. DEX refers to dexamethasone, 25 μg/mL. Data are presented as means ± SD (n = 3, biological replicates). “mM” means mmol/L; “μM” means μmol/L. *** *p* < 0.001.

Based on previous methods [27], the cytotoxicity of DESP on iBMDM macrophages was evaluated using the CCK-8 assay. The CCK-8 reagent is reduced to a colored substance by dehydrogenases in live cell mitochondria, and its absorbance (450 nm) is proportional to the number of live cells [58]. As shown in Figure 9, DESP showed no cytotoxic effect on iBMDM macrophages at concentrations ranging from 25 to 300 μg/mL.

#### 3.6.2. Effect of DESP Concentrations on Cytokine Secretion

Cytokines are known to play significant roles in pro-inflammatory responses. Inhibiting the release of pro-inflammatory cytokines is a potential strategy adopted by many anti-inflammatory drugs for treating inflammation [58]. Our previous research found that sweet potato pectin showed good anti-inflammatory activity [27]. For the anti-inflammatory action assessment of DESP, the tumor necrosis factor -α (TNF-α), interleukin-1 (IL-1β), and interleukin-6 (IL-6) outputs in LPS-stimulated iBMDM macrophages were explored. Figure 9 indicates the successful establishment of an inflammatory model. After a treatment of DESP (75 μg/mL), the concentrations of IL-1β, IL-6, and TNF-α decreased by 4.73 ± 0.11 ng·mL^−1^, 3.68 ± 0.20 ng·mL^−1^, and 2.16 ± 0.14 ng·mL^−1^, respectively. As we know, the natural polysaccharide possesses anti-inflammatory effects in vitro by suppressing the generation of pro-inflammatory cytokines; similar results were obtained with DESP in this work.

### 3.7. Comparison of the Effects Between the DES Extraction and Traditional Extraction

In order to more intuitively evaluate the advantages of DES extraction in improving SPR extraction efficiency, a comparison was made between DES extraction, hot water extraction, and hydrochloric acid extraction of SPR. As shown in Table 7, the extraction yield of the DES extraction method used in this study (5.6%) was significantly higher than for other extraction methods. However, the DES method requires more extraction time (125 min) compared to the conventional solvent method [27]. In the future, we will investigate the use of ultrasound-assisted DES to extract SPR and explore whether it can shorten the extraction time.

## 4. Conclusions

This work proved that choline chloride–urea was an effective extraction solvent with which to achieve a higher yield of sweet potato residue pectin (DESP). DESP was extracted under the optimum conditions suggested by RSM: water content of 75 wt.%, extraction time of 125.7 min, extraction temperature of 83.2 °C, and a liquid-to-solid ratio of 37.0 mL·g^−1^. The optimized extraction yield was 5.6% ± 0.09%, which was 2.4 times higher than that of HWSP. In addition, the preliminary structure of DESP was analyzed by FT-IR, XRD, and SEM analyses. The *M*w of DESP was 20.90 kDa, which was lower than that of HWSP. The monosaccharide composition of DESP was similar to that of HWSP and mainly included galacturonic acid, glucose, galactose, arabinose, rhamnose, and mannose. However, the content of GalA in DESP was significantly lower than in HWSP, while the content of Glc was significantly higher. In addition, DESP exhibits certain inhibitory effects on the production of IL-1 β, IL-6, and TNF-α.

## Figures and Tables

**Table 1:** The types of DESs.

Abbreviation	HBA	HBD	Molar Ratio
DES-1	Choline chloride	1,3-Propylene glycol	1:2
DES-2	Choline chloride	Glycerol	1:2
DES-3	Choline chloride	Citric acid	1:4
DES-4	Potassium carbonate	Glycerol	1:4
DES-5	Choline chloride	Urea	1:2
DES-6	Choline chloride	1,4-Butanediol	1:2

**Table 2:** Factors and levels of RSM analysis.

Independent Variables	Levels		
	−1	0	1
A DES water content (wt. %)	45	60	75
B Extraction time (min)	90	120	150
C Extraction temperature (°C)	60	80	100
D Liquid–solid ratio (mL·g^−1^)	25	35	45

**Table 3:** BBD and results of the yield of sweet potato pectin.

Run	A wt.%	B min	C °C	D mL·g^−1^	Y/%
1	60	120	60	25	4.37
2	75	90	80	35	4.19
3	75	120	100	35	5.01
4	60	90	100	35	4.69
5	75	120	80	45	5.32
6	60	90	80	25	3.43
7	60	90	60	35	3.30
8	60	120	80	35	5.59
9	75	120	80	25	4.77
10	45	120	60	35	3.89
11	60	150	80	25	5.39
12	75	120	60	35	4.61
13	45	120	80	45	3.70
14	45	90	80	35	3.99
15	45	120	80	25	5.19
16	60	120	80	35	5.69
17	60	120	60	45	3.51
18	60	120	100	45	4.65
19	60	120	80	35	5.87
20	60	150	80	45	3.88
21	75	150	80	35	5.38
22	45	120	100	35	4.45
23	60	150	60	35	4.22
24	60	150	100	35	4.33
25	60	120	80	35	5.99
26	60	120	100	25	4.57
27	60	90	80	45	4.43
28	60	120	80	35	5.59
29	45	150	80	35	4.48

**Table 4:** Variance analysis of the regression model.

Source	Sum of Squares	df	Mean Square	F Value	*p*-Value Prob > F	Significant
Model	15.54	14	1.11	41.16	<0.0001	***
A	1.07	1	1.07	39.61	<0.0001	***
B	1.11	1	1.11	41.17	<0.0001	***
C	1.20	1	1.20	44.63	<0.0001	***
D	0.41	1	0.41	15.37	0.0015	**
AB	0.12	1	0.12	4.54	0.0513	
AC	0.01	1	0.01	0.24	0.6337	
AD	1.04	1	1.04	38.58	<0.0001	***
BC	0.41	1	0.41	15.19	0.0016	**
BD	1.58	1	1.58	58.41	<0.0001	***
CD	0.22	1	0.22	8.19	0.0125	*
A^2^	1.07	1	1.07	39.80	<0.0001	***
B^2^	4.31	1	4.31	159.98	<0.0001	***
C^2^	4.46	1	4.46	165.42	<0.0001	***
D^2^	2.56	1	2.56	94.87	<0.0001	***
Residual	0.38	14	0.03			
Lack of Fit	0.25	10	0.03	0.79	0.6534	
Pure Error	0.13	4	0.03			
Cor Total	15.92	28				
R^2^	0.9763					
R^2^_adj_	0.9526					
R^2^_pred_	0.8968					
Adeq precision	21.89					
C.V.%	3.54					

* *p* < 0.05; ** *p* < 0.01; *** *p* < 0.001.

**Table 5:** The yields and *M*w of DESP.

Samples	DESP	HWSP [27]	HASP [27]
Yields (%, *w*/*w*)	5.6	2.3	2.1
*M*n (kDa)	17.61	39.25	51.97
*M*w (kDa)	20.90	58.41	69.81

**Table 6:** Monosaccharide components of DESP (mol %).

Pectin Samples	DESP	HWSP [27]	HASP [27]
Chemical composition			
Total sugar content (%, *w*/*w*)	92.8 ± 1.21	91.9 ± 1.64	91.5 ± 1.77
Monosaccharide (mol %)			
Rha	4.13 ± 0.40	10.49 ± 0.18	11.04 ± 0.45
Ara	12.10 ± 0.85	8.58 ± 0.50	2.16 ± 0.13
Gal	17.90 ± 0.20	33.62 ± 1.41	22.62 ± 0.94
Glc	25.63 ± 0.76	6.12 ± 0.75	6.46 ± 0.46
Xyl	1.07 ± 0.12	0.74 ± 0.12	0.99 ± 0.05
Man	0.57 ± 0.058	0.49 ± 0.070	0.66 ± 0.11
GalA	47.07 ± 1.58	41.50 ± 0.82	54.13 ± 0.75
GlcA	-	1.43 ± 0.13	1.41 ± 0.07
HG/%	42.93 ± 1.40	31.07 ± 0.65	43.08 ± 0.72
RG-I/%	38.27 ± 1.71	63.19 ± 1.34	46.87 ± 1.89
(Ara + Gal)/Rha	7.26 ± 0.57	4.02 ± 0.05	2.25 ± 0.05
DE/%	26.15	47.48	14.26

Note: Total sugar content was detected by phenol–H_2_SO_4_ method. RG−I = [GalA − HG] + Rha + Ara + Gal; HG: homogalacturonan; HG = GalA − Rha.

**Table 7:** Comparison of the effects of DES extraction and traditional extraction.

Extraction Method	Yield (%, *w*/*w*)	*M*n (kDa)	Time (min)	Reference
Hot water extraction	2.3	39.25	90	[27]
Hydrochloric acid extraction	2.1	51.97	90	[27]
DES extraction	5.6	17.61	125	This work

## Data Availability

The original contributions presented in the study are included in the article and Supplementary Materials, further inquiries can be directed to the corresponding authors.

## Supplementary Materials

The following supporting information can be downloaded at: https://www.mdpi.com/article/10.3390/foods15020388/s1, Table S1: All ELISA original data.
