# Supplementary material for: Deep Eutectic Solvents Mediated Extraction of a Pectin Polysaccharide from Processed Sweet Potato By-Products: Optimization and Characterization Studies
Source: Foods. 2026 Jan 21;15(2):388. doi: 10.3390/foods15020388 (PMC12841006; doi:10.3390/foods15020388)
Supplement: Supplementary file 1 [file foods-15-00388-s001.zip › foods-4068415-supplementary.pdf]

Supplementary documents

Ms. Ref. No.: **foods-4068415**

Title: Deep eutectic solvents mediated extraction of a pectin poly-saccharide from processed sweet potato by-products: Optimization and characterization studies

1. Mw determination and monosaccharide composition analysis

The weight–average molecular weight (*M*w) and number–average molecular weight (*M*n), of the pectins were determined using high-performance size exclusion chromatography coupled to a multiangle laser light scattering refractive index (HPSEC–MALLS–RI, λ=658 nm; Wyatt Technologies Corporation, USA). HPSEC–MALLS was performed on an LC-10A Shimadzu HPLC system (Shimadzu, Japan) equipped with a Shimadzu RI-10A refractive index detector (RID, Japan) and a BRT105-103-101 SEC column (8 × 300 mm, BoRui Saccharide) in a series at 25 °C. A total of 0.05 M NaCl was used as the elution solvent at a flow rate of 1.0 mL/min. Moreover, the pectin samples were dissolved in this solvent at 1.0 mg/mL and filtered through 0.45 μm membranes prior to injection. The dn/dc value used was 0.1380 mg/L. The light scattering model was the Zimm model. The on-line ASTRA 8.1 software package (Wyatt Technologies, USA) was used for data analysis.

High-performance anion-exchange chromatography (HPAEC) using a pulsed amperometric detector (PAD) equipped with a Dionex CarboPac PA- 20 anion-exchange column (3 × 150 mm, 10 μm) was used to test the monosaccharide composition of the pectins. The pectin samples (5 mg) were hydrolyzed with trifluoroacetic acid (TFA, 2 mL, 3 M) at 120 ℃ for 3 h, followed by transferring to the tube for nitrogen purging and drying. 5 mL water was added and mixed well, then 100 µL aqueous solution was added to 900 µL of deionized water and centrifuged at 12000 rpm for 5 minutes. Finally, the treated pectin solutions (5 µL) were introduced into the system and gradient eluted by mobile phases A (H_2O), B (250 mM NaOH) and C (500 mM NaOH & 50 mM NaAc) at a flow rate of 0.3 mL/min. The standard monosaccharides include fucose, rhamnose, arabinose, galactose, glucose, xylose, fructose, galacturonic acid, glucuronic acid, and mannose

2. All ELISA original data

Table S1

Cell viability（%）	0	25(μg/mL)	75(μg/mL)	100(μg/mL)	150(μg/mL)	300(μg/mL)
	99.8697	96.8901	98.8236	104.5235	103.9468	101.8567
	100.3464	96.1513	100.6257	101.5365	101.7377	102.5758
	99.6797	97.3546	102.8664	103.4855	102.7567	106.5576
IL-1β (ng/mL)	ctrl	LPS	DEX	DESP(75 μg/mL)	DESP(150 μg/mL)	DESP(300 μg/mL)
	0.3219	6.9346	0.4980	4.6346	2.6580	1.9639
	0.3427	6.8637	0.5320	4.8463	2.7956	1.9197
	0.3641	6.6359	0.5110	4.6978	2.8564	1.8965
IL-6 (ng/mL)	ctrl	LPS	DEX	DESP(75 μg/mL)	DESP(150 μg/mL)	DESP(300 μg/mL)
	0.2925	4.7589	0.5328	3.8749	2.8750	1.7549
	0.2858	4.7408	0.5905	3.6947	2.9754	1.6438
	0.2883	4.5139	0.6298	3.4770	2.5398	1.5986
TNF-a (ng/mL)	ctrl	LPS	DEX	DESP(75 μg/mL)	DESP(150 μg/mL)	DESP(300 μg/mL)
	0.1892	2.8304	0.6935	2.0069	1.1090	0.9467
	0.1973	2.8308	0.7246	2.2787	1.2247	0.9037
	0.2341	2.6947	0.6846	2.1897	1.2848	1.1247
